# Supporting peer researchers: recommendations from our lived experience/expertise in community-based research in Canada

**DOI:** 10.1186/s12954-019-0322-6

**Published:** 2019-09-03

**Authors:** Francisco Ibáñez-Carrasco, James R. Watson, James Tavares

**Affiliations:** MAP Centre for Urban Health Solutions, St Michael’s Hospital, Toronto, ON Canada

**Keywords:** Peer researchers, Community-based research, Best practices for support, Lived experience of HIV

## Abstract

Community-based research in HIV in Canada is a complex undertaking. Including peer researchers living with HIV meaningfully is intricate and costly. However, this inclusion guarantees results that translate to community action, policy-making, and public awareness. Including HIV+ peer researchers expedites the path from research to intervention. However, we must constantly review our support in light of three implicit tasks performed by peer researchers: constant disclosure, emotional labor, and advocating for meaningful participation. Our team offers four pillars of support to reduce harm and strengthen the self-determination, confidence, advocacy, and impact for HIV+ peer researchers. The provision of emotional, instrumental, educational, and cultural/spiritual support might seldom be standardized within a study, but to successfully engage in community-based research, study teams must articulate what support can be offered in each area.

Community-based research (CBR) in Canada came into maturity during the AIDS epidemic. In tandem with researchers, peer researchers (PRs) living with HIV/AIDS, pushed the scope and boundaries of biomedical and social-behavioral studies on AIDS. Three decades later, peer research is acknowledged by scientific research funders including the Canadian Institutes of Health Research’s (CIHR) Strategy for Patient Oriented Research, launched in 2014. This acknowledgement, in turn, affirms the power of patient contributions to forge an expedited, streamlined path from research and intervention [1]. Despite these advances, we are still learning how best to support our peer researchers as they draw from and share their lived experience within a structured research project. Ibáñez-Carrasco has been living with HIV/AIDS for 35 years; he obtained his doctorate in education 15 years after becoming a peer researcher. Watson has been living with HIV/AIDS for 25 years and began his career as peer researcher in a provincial study of health, HIV/AIDS, and housing. We have worked together since 2009. In this journal article, we share key lessons learned. In some cases, we are undertaking research to evaluate our claims. In others, existing literature supports our lived experience.

Our definition of who a peer researcher (PR) is and their role within a research team is broad and includes many variations to reflect how the role is practiced in HIV/AIDS health research across Canada. A PR is a “patient,” a lay person living with HIV/AIDS. They are not schooled or accredited in research and may participate in one or all phases of a project. A PR can be a member of a research project’s advisory board, a data collector, or a contributor in the knowledge transfer and exchange (KTE) process. When PRs are given appropriate support, their contributions include critical examination and intimate understanding of the motives, processes, and outcomes of a CBR project [2].

In our experience, PRs are encumbered with three implicit tasks in addition to their technical duties: disclosing their HIV positive status, engaging in emotional labor, and striving and advocating for meaningful involvement in their work. These tasks can be onerous and even harmful. It is our duty to reduce the potential harms.

## Constant disclosure of HIV positive status

First, constant disclosure is necessary for a PR to claim “lived experience” with HIV/AIDS. “Lived experience” is to have personal and intimate knowledge and practice of living with HIV/AIDS. This includes facing social stigma and the tribulations of having an episodic condition with intermittent periods of illness and wellness. In the context of CBR, the concept and practice of “disclosure”—verbally or through any media—precedes a PR employment with a research project. We expect our PRs to externalize, reflect upon, interpret, and apply their lived experience to their work. Meanwhile, we as researchers do not always know how to best support PRs as they deploy their lived experience in the communities they belong to, cope with the emotions that accompany a study’s end, or face the consequences of increased visibility in their community.

## Engaging in emotional labor

Second, a degree of “emotional labor” always accompanies disclosure because telling others one is HIV positive is strategic and compassionate. Emotional labor is “the work that people do in ‘performing’ feelings, including through regulating and managing practices in this area, within normative organizational and social contexts; and as the ‘work’ that these practices do in constructing and reflecting social realities, identities, relations and institutions — and thus social structures (including those of gender and social class)” (p. 281) [3]. Though a singular, universal patient living with HIV/AIDS does not exist, PRs are expected to be highly attuned to persons living with HIV from all walks of life.

## Striving and advocating for meaningful involvement in their work

Thirdly, in our experience, PRs are often called upon, explicitly or implicitly, to advocate for HIV/AIDS to be accommodated as a chronic and episodic health condition, and to advocate for the uniqueness of living with a stigmatized and sometimes criminalized condition. Acts of advocacy are often performed in addition to the daily stress of living as a member of an ethno-racialized and sexual/gender minority. Thus, advocating for the observance of our HIV-positive right to self-determination, the Greater/Meaningful Involvement of People Living with HIV/AIDS (GIPA/MIPA)—a set of principles adopted globally by governance of persons living with HIV [4]—often falls disproportionally upon PRs.

## Supporting peer researchers: four recommendations

It is our duty to reduce harms inflicted on PRs by the research industry. As peer researchers manage these three implicit tasks as well as performing the technical research tasks associated with their role, study investigators must consider what supports they offer to working PRs. In our research projects, we deploy four types of support: emotional and identity, instrumental, educational, and cultural/spiritual. These supports are rooted in the philosophy and tenets of CBR and harm reduction and, as such, strive to be empowering, equitable, and accessible [5].

## 1. Emotional and identity support

Providing lived experience in the context of a research project triggers difficult emotions in PRs. As a result, they may have trouble separating their own unique lived experience from that of study participants. PRs may also experience difficult emotions or even depression, after a study ends along with the withdrawal of support, structure, and process provided by a research team. Further, PRs may experience peer burnout as well as the stress and pressure associated with serving as a role model to peer participants.

However, focusing only on negative impacts on the PR can perpetuate a culture of victimhood. Research work and being a role model to individuals and communities can also strengthen the PR’s self-esteem, professional achievement, confidence, and altruism. The difficulties of disclosure, emotional labor, and advocacy must be considered in light of PRs’ resilience, their ability to theorize from the ground up, and their impact in guiding a team (e.g., describing to the research team how things are done in a specific milieu) [6].

### Recommendations

To properly support PRs, research teams must prepare them for the ongoing shifts in role, identity, and experience before the fieldwork begins. This goes beyond having a clear set of expectations and terms of reference. During a study, PRs should receive communication and conflict management skills, ongoing counseling, further professional opportunities in and out of HIV/AIDS research, and access to spiritually and culturally relevant support services. For example, at the MAP Centre for Urban Health Solutions, our PR support practices include the “Essential Tools for Support and Stability Peer Research ToolKit” developed at the AIDS Bereavement and Resiliency Program of Ontario [7]. At the time of this writing, we are collaborating to assess the Toolkit efficacy with a team of PRs involved in the Canadian version of the HIV/AIDS Stigma Index.

## 2. Instrumental support

CBR studies often grapple with systemic social inequities and the people most impacted by them. Oftentimes, PRs are themselves members of vulnerable populations and possess an intimate understanding of their community’s politics, social malaise, HIV/AIDS stigma, racism, homophobia, and substance use. Investigators facilitate the logistic and administrative tasks which are often fairly bureaucratic in academic institutions. Instrumental support should include clear and accessible research plans, communication and reporting procedures, technical guidelines (e.g., how to troubleshoot electronic devices used in data collection), and administrative forms (e.g., for reimbursements). They should be written in a balanced combination of plain and technical language and accessible using accessible media (e.g., paper, smartphones). Instrumental support includes making workplace accommodations congruent with an episodic disability and with substance addictions (e.g., working evenings, having unscheduled rest, etc.).

Compensation and payment modalities also matter a great deal. Canadian institutions have begun implementing compensation guidelines such as the guidelines proposed by Greer et al. [8]. These include outlining clear procedures for paying peers, knowing about the income exemptions for peers on disability or income assistance, and strategies for overcoming barriers to equitable pay. Nonetheless, disbursing monies to persons in low socio-economic strata and receiving governmental support and subsidies brings complications. To attenuate these problems, and not always mentioned in formal documents, researchers sometimes offer honoraria as gift cards (which are not reportable as earnings), make payments in cash (for PRs who do not have bank accounts), and/or stagger payments over weeks or months regardless of when income was earned. However, this is toeing prickly institutional lines, not all institutions would consider these strategies permissible. Further advocacy work and research are required from CBR practitioners within academia.

### Recommendations

We recommend including PRs in the financial planning of CBR projects, prior to official employment, to establish flexible payment plans tailored to their economic situation. We also recommend choosing work titles wisely because CBR is practiced within hierarchical research institutions. The title given to a PR orients them within that hierarchy and may put at ease grant reviewers and members of Review of Ethics Board members (REB) who continue to exhibit unfamiliarity with CBR approaches. Properly naming the work and role of PRs attenuates the dissonance between their public identification (e.g., “I work for a research study, institution, or researcher”) and their institutional identification (e.g., I am a “small vendor,” “consultant,” “peer researcher,”), and it somewhat balances the power differential between the academic and the community personnel [9].

## 3. Educational support

We notice that the uncritical training of patients as peer researchers tends to develop specialized individuals but does not prepare peers for leadership, civic life, or permanent employment. Those leadership attributes and practices are often intuitively *picked up* along the research way. In this regard, PR training may just sustain chronic underemployment, and a gig economy, as it is limited to specific tasks devoid of critical perspective. Working as a PRA for only specific tasks such as data collection inhibits decision-making power within the broader research process, thus producing a phenomenon tantamount to the Marxist concept of *deskilling*.

### Recommendations

Research teams should critically examine whether they are training PRs for the task or for the future. PRs indeed have to learn to perform tasks such as seeking informed consent, interviewing, and data analysis. Additionally, PRs must also be trained and supported to become opinion leaders, ethics champions, future trainers, and employable individuals. Training should include building skills (know-how) and reflection (know-why). In our experience, holistic training varies considerably from one study to another but efforts should be made to provide frequent and ongoing learning opportunities in-person or online. Most importantly, these efforts must be evaluated and communicated to PRs, not in a schooling or labor performance evaluation manner, but in summative ways. These would truly help a PR see progress and identify areas of growth.

In our research, we measure PRs learning of training concepts and activities and assess individual PR’s comfort with their role. We also adapt study-specific curriculum to the emergent conditions of a study and provide timely feedback to the PRs. Our learning opportunities apply a blended approach delivered in-person and online using synchronous and asynchronous digital modules and podcasts to facilitate individual and group study. Our e-learning modules are built in collaboration with, and are funded by, PRs, nonprofit leaders, and academics (See Universities Without Walls www.uwow.ca).

In our practice, to evaluate PR’s learning, we use a pre- and post-intervention assessment instrument adapted from Brocklehurst and Rowe [10] to measure awareness/knowledge as well as skills and experience using retrospective pre and post self-ratings, thus mapping out before-and-after responses. Each learner rates themselves on a continuum from “Novice” to “Expert” in the areas of knowledge, skills, and experience.

## 4. Cultural/spiritual support

Research studies may collect powerful data and influence practice and policy but they can also leave PRs feeling unseen and unacknowledged. A study team must support and affirm the intersectionality of PRs and include knowledge and practice of Indigenous research, gender, diversity, social justice, and anti-oppressive methodology. One example is our focus on the cultural and spiritual support to Indigenous PRs in HIV/AIDS research as well as our support of minorities and newcomers also involved in HIV/AIDS research. The Canadian Truth and Reconciliation Commission report compels us to conduct research honoring and protecting Indigenous children, incarcerated Indigenous peoples, Indigenous women, and respectful “models of research and treatment in which Aboriginal communities have ownership, control, access, and possession” [11] (p. 208).

### Recommendations

Due to the ongoing impact of Western colonization, researchers must consider the forces that continue to shape Indigenous people’s day-to-day experience. Elders and Indigenous PRs (including two-spirit PRs) bring a “two-eyed seeing” perspective encompassing in equal valuation both Western and Indigenous knowledge. It is important to observe Indigenous ceremony to ensure research is conducted “in a good way”. Researchers must also recognize that the Indigenous diaspora in Canada includes a range of beliefs and ceremony that are not homogenous and that diverse Indigenous traditions, practices, and cultures are unique to each community and nation.

Paying close attention to cultural and spiritual practices may seem cumbersome and peripheral to the métier of research. However, the operationalization of “cultural safety” and “trauma-informed” activities halts the reproduction of colonial Western patriarchal research. Researchers should take the time to understand and honor the unique identities and spiritualities within their team before deploying PRs and research tools into the field.

## Conclusion

CBR has become a very complex undertaking. Including PRs meaningfully is indeed both difficult and costly. However, CBR with PRs guarantees meaningful results that translate to community action, policy-making, and public awareness and can expedite the journey from research to intervention. In addition, implementation of our four pillars of support strengthens the self-determination, confidence, advocacy, and impact for PRAs. We do not foresee a future in health research without the direct and meaningful participation of the “patients.” The supports recommended here are evolved from our combined lived experience and a broad reading of the academic and gray literature in the field. The provision of emotional, instrumental, educational, and cultural/spiritual support might seldom be standardized within a study, but to successfully engage in community-based research, study teams must articulate what support can be offered in each area.

## Data Availability

Data sharing is not applicable to this article as no datasets were generated or analyzed during the current study.

## Abbreviations

PR: Peer researcher
HIV/AIDS: Most commonly used combined acronym for the virus, conditions, and opportunistic illness caused by the human immunodeficiency virus
CBR: Community-based research (CBR) is used in Canada more often than community-based participatory research (CBPR), both acronyms should be understood as interchangeable in this manuscript
HAND: HIV/AIDS-associated neurocognitive disorders (HAND)
The GIPA/MIPA principles: (Greater/Meaningful Involvement of People Living with HIV/AIDS). GIPA is a guiding principle that calls for the active and meaningful participation of people living with HIV/AIDS in the inception, development, implementation, monitoring, and evaluation of policies and programs
